# Supplementary material for: SLAM-seq reveals independent contributions of RNA processing and stability to gene expression in African trypanosomes
Source: Nucleic Acids Res. 2024 Dec 14;53(3):gkae1203. doi: 10.1093/nar/gkae1203 (PMC11797058; doi:10.1093/nar/gkae1203)
Supplement: gkae1203_Supplemental_Files [file gkae1203_supplemental_files.zip › Luzak_supplementary_data.pdf]

Supplementary Figures

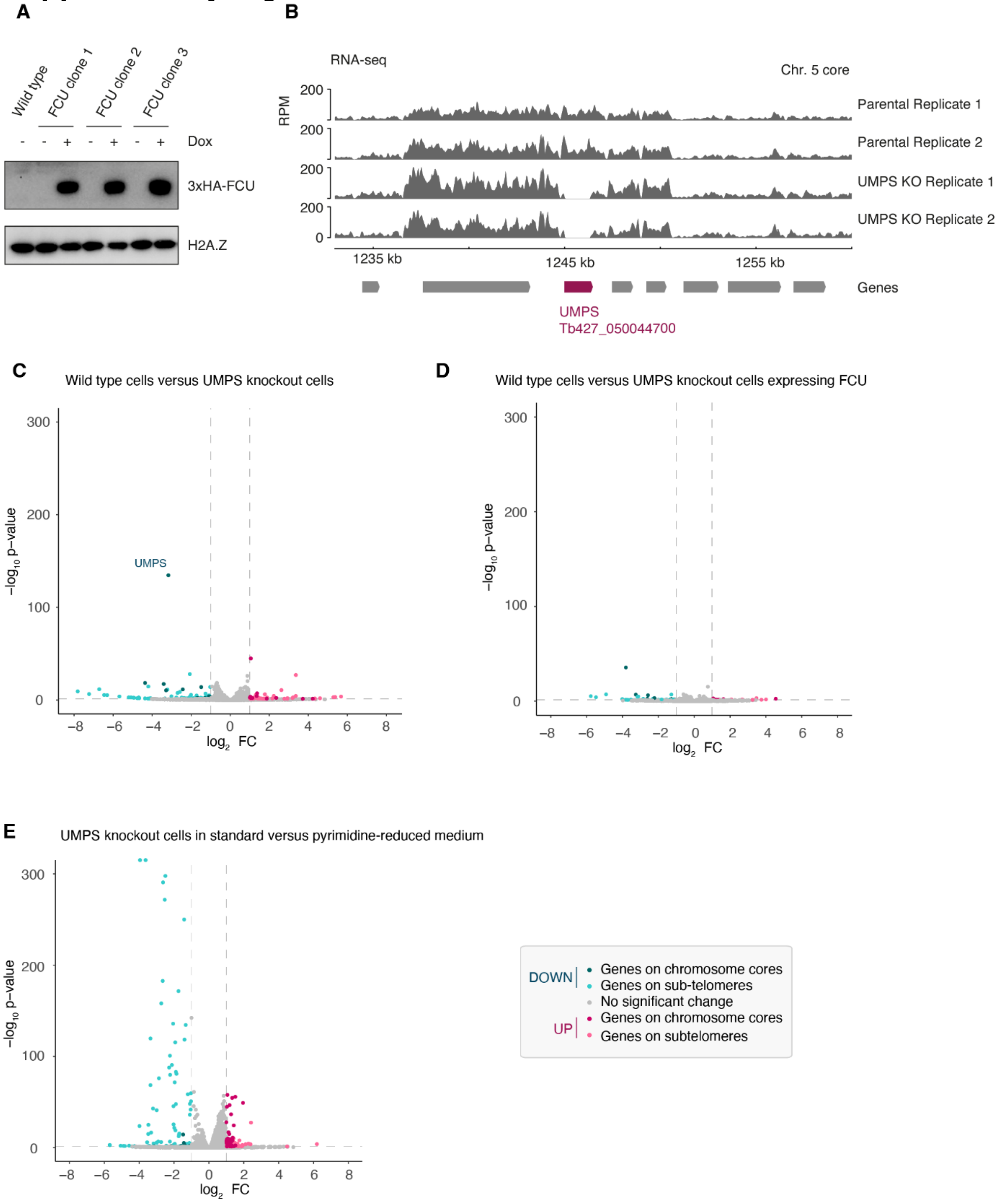

Supplementary Figure S1. Confirmation of exogenous FCU expression and *UMPS* knockout. **(A)** FCU expression was induced for 24 hours with doxycycline and expression was confirmed by western blot analysis in three independent clones. **(B)** UMPS double knockout was verified by RNA-seq analysis in wild-type and UMPS KO cells. **(C)** Differential gene expression in wild-type versus UMPS KO cells was measured by RNA-seq. The Volcano plot shows unchanged genes in grey, upregulated genes in pink and downregulated genes in green (n=3). **(D)** Differential gene expression in wild-type versus UMPS KO cells expressing FCU was measured by RNA-seq. The Volcano plot shows unchanged genes in grey, upregulated genes in pink and downregulated genes in green (n=3). **(E)** Differential gene expression in UMPS KO cells in standard versus pyrimidine-reduced medium was measured by RNA-seq. Cells were incubated or 15 minutes in either standard or pyrimidine-reduced medium prior to RNA harvest. The Volcano plot shows unchanged genes in grey, upregulated genes in pink and downregulated genes in green (n=3).

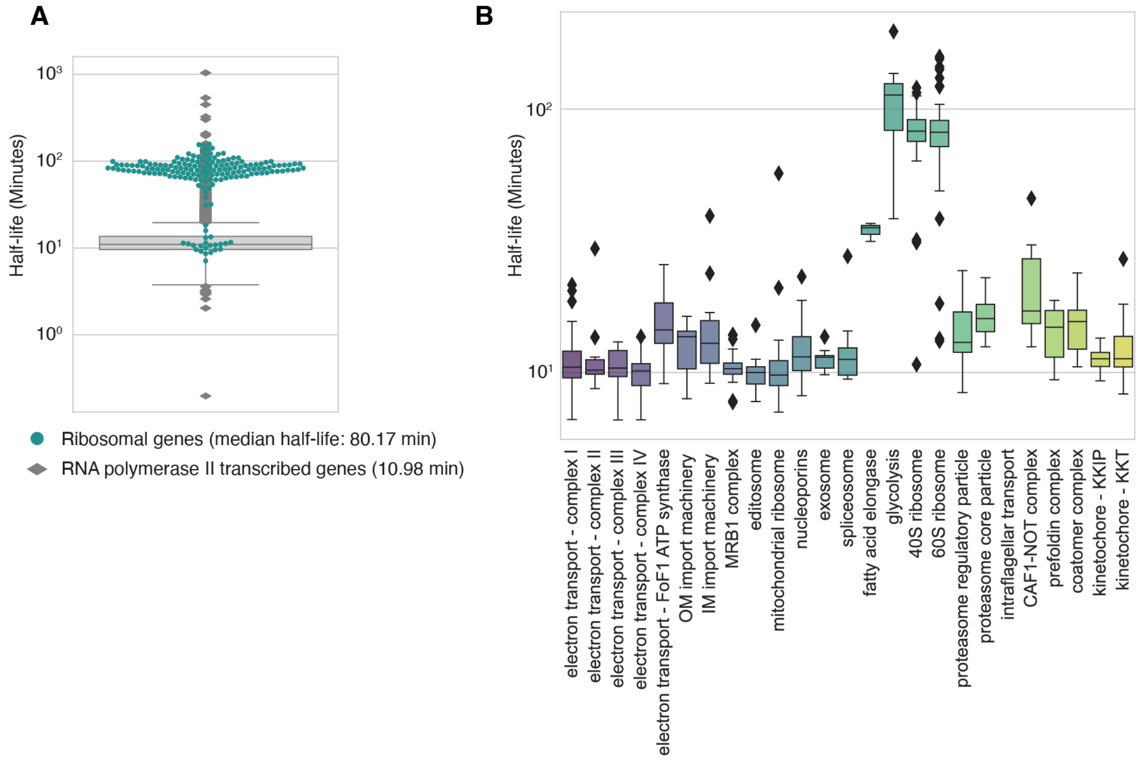

Supplementary Figure S2. mRNA half-lives are co-regulated for components of functional protein complexes. (**A**) RNA half-lives determined by SLAM-seq are plotted for RNA polymerase II transcribed genes as box blots, including ribosomal genes. The box represents 25-75 % of the data points, the bar represents the median for the underlying data, the whiskers represent 1.5*IQR and the rhombi represent outliers. On top of the box plot, mRNA half-lives of transcripts encoding ribosomal proteins are further highlighted as individual dots. (**B**) RNA half-lives determined by SLAM-seq are plotted as box plots for mRNAs encoding components of protein complexes. The box represents 25-75 % of the data points, the bar represents the median for the underlying data, the whiskers represent 1.5*IQR and the rhombi represent outliers. Experimentally validated protein complexes were extracted from Moloney *et al.*, 2023 (76).

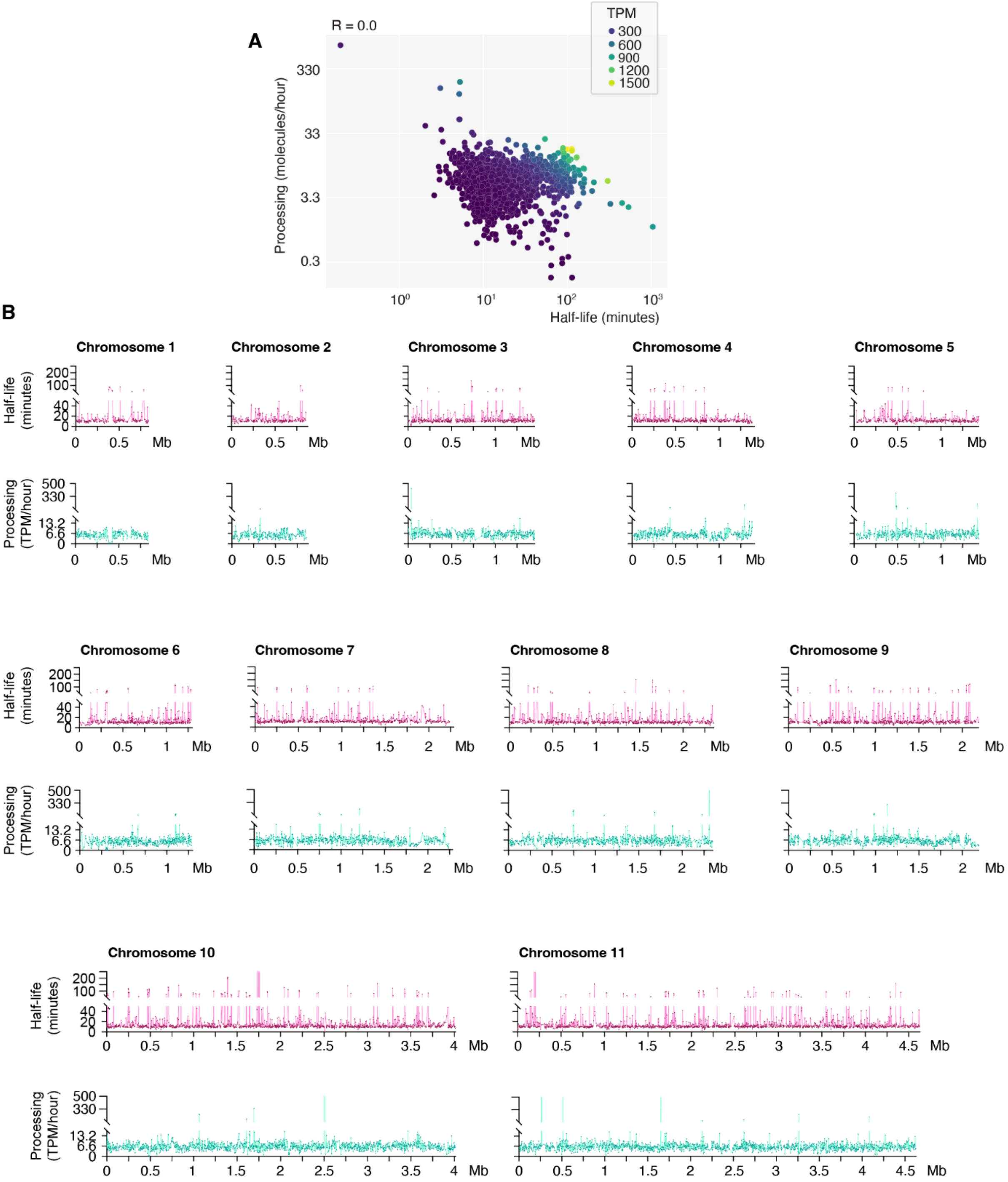

Supplementary Figure S3. Genome-wide distribution of RNA processing and stability measurements in *T. brucei*. **(A)** Correlation analysis of RNA half-lives and RNA processing rates, both measured by SLAM-seq. Each dot represents a gene. Dots are colored according to the respective RNA total level of the gene. **(B)** RNA processing rates and half-lives were determined for all RNA polymerase II transcribed genes by SLAM-seq and plotted along the 11 megabase chromosome cores of *T. brucei*.

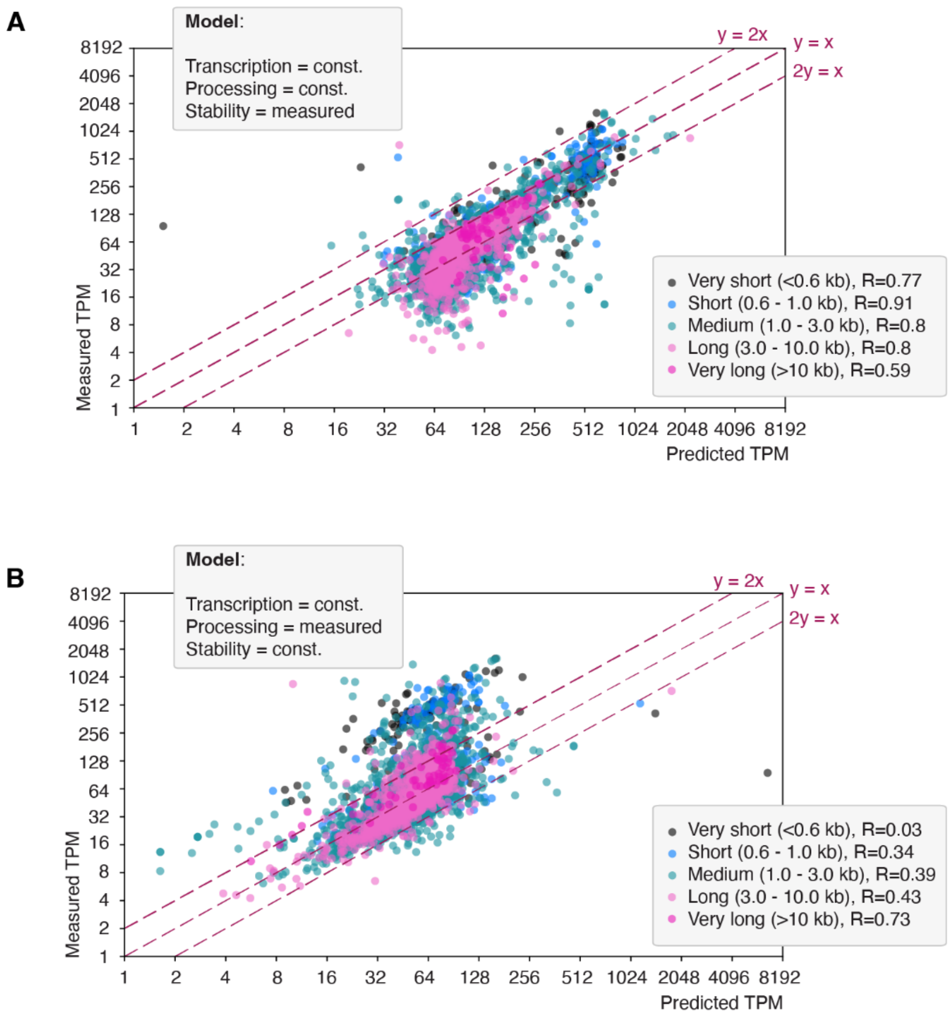

Supplementary Figure S4. Transcript length determines how strongly genes are affected by mRNA processing efficiency. **(A)** Correlation of predicted and measured total mRNA levels. Prediction of total levels was performed assuming constant transcription and constant RNA processing rate. Measured half-lives were introduced in a gene-specific manner. Genes were grouped according to transcript length. **(B)** Correlation of predicted and measured total mRNA levels. Prediction of total levels was performed assuming constant transcription and constant RNA stability. Measured processing rates were introduced in a gene-specific manner. Genes were grouped according to transcript length.

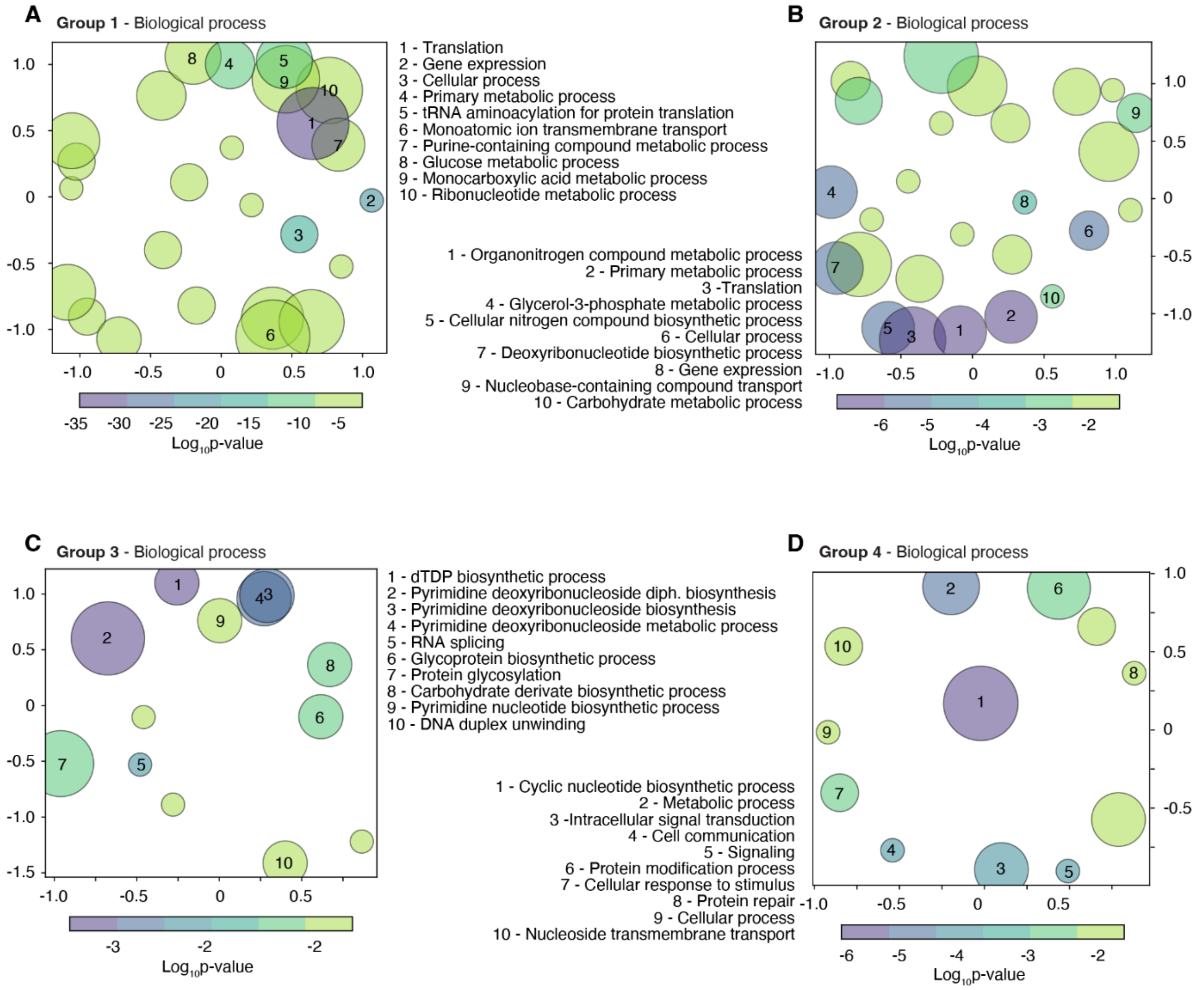

Supplementary Figure S5. GO-term analysis reveals distinct biological processes represented in four gene groups. Genes were classified into four regulatory groups according to their RNA stability and processing rates. **(A)** GO-term analysis for enrichment of biological processes was performed for 615 genes in group 1 with highest half-lives and highest processing rates. **(B)** GO-term analysis for enrichment of biological processes was performed for 411 genes in group 2 with highest half-lives and lowest processing rates. **(C)** GO-term analysis for enrichment of biological processes was performed for 402 genes in group 3 with lowest half-lives and highest processing rates. **(D)** GO-term analysis for enrichment of biological processes was performed for 323 genes in group 4 with lowest half-lives and lowest processing rates.

Reference

76. Moloney,N.M., Barylyuk,K., Tromer,E., Crook,O.M., Breckels,L.M., Lilley,K.S., Waller,R.F. and MacGregor,P. (2023) Mapping diversity in African trypanosomes using high resolution spatial proteomics. *Nat. Commun.*, **14**, 4401.
