# Supplementary material for: SLAM-seq reveals independent contributions of RNA processing and stability to gene expression in African trypanosomes
Source: Nucleic Acids Res. 2024 Dec 14;53(3):gkae1203. doi: 10.1093/nar/gkae1203 (PMC11797058; doi:10.1093/nar/gkae1203)
Supplement: gkae1203_Supplemental_Files [file gkae1203_supplemental_files.zip › Supplementary_Table_S1_24-11-18.docx]

**Supplementary Table S1: Relevant sequences used for cell line generation**

| **Name** | **Sequence** |
| --- | --- |
| GB37_FCU_CDS | AATTACACCAAAAAGTAAAATTCACAAGCTT**ATG**GTAACTGGTGGCATGGCGTCCAAGTGGGACCAGAAGGGGATGGATATCGCTTATGAGGAAGCCGCATTAGGGTACAAAGAAGGAGGCGTGCCTATTGGTGGCTGTCTTATAAATAACAAGGATGGGAGCGTATTGGGCCGGGGACATAACATGCGCTTCCAGAAAGGTAGTGCAACATTGCACGGCGAGATTTCGACTCTGGAAAACTGCGGACGCTTGGAAGGTAAGGTGTATAAAGACACCACTCTCTACACTACACTTAGCCCTTGCGATATGTGCACCGGAGCGATAATTATGTATGGTATTCCGCGTTGTGTTGTTGGGGAAAATGTCAACTTTAAGAGCAAAGGTGAAAAATATTTGCAAACACGGGGGCATGAAGTGGTTGTAGTTGATGACGAACGTTGCAAGAAGATTATGAAACAGTTTATCGACGAGAGACCACAGGACTGGTTCGAAGATATCGGTGAGGCTTCGGAGCCTTTCAAGAACGTGTATTTACTGCCCCAGACGAACCAACTTTTGGGTCTTTACACGATCATTAGGAATAAAAATACGACACGACCGGACTTTATCTTTTACAGTGATCGCATAATTAGGCTGCTCGTGGAAGAGGGACTCAATCACTTGCCAGTTCAAAAGCAAATAGTTGAGACCGATACAAACGAAAATTTCGAGGGCGTGTCATTCATGGGAAAAATCTGTGGTGTATCCATTGTGCGAGCGGGGGAGTCAATGGAACAAGGACTACGTGACTGTTGCCGGTCTGTCAGGATTGGCAAAATACTTATTCAGAGAGATGAAGAGACGGCCCTTCCGAAACTGTTTTATGAGAAGCTCCCGGAGGACATTTCTGAACGTTATGTCTTTCTCCTAGATCCAATGCTTGCGACCGGGGGATCAGCTATAATGGCAACGGAGGTGTTAATTAAACGTGGTGTTAAGCCAGAGCGCATCTACTTTCTGAACCTGATATGCAGTAAGGAAGGGATCGAGAAGTACCACGCAGCTTTCCCCGAGGTGCGAATTGTCACAGGTGCCCTAGATCGGGGCTTGGACGAGAATAAGTACCTTGTGCCCGGTCTCGGTGATTTTGGAGATCGCTACTACTGTGTT**TGA**CTCGAGGGATCCTGCCCATTTAGTTGGCTT |
| N-terminal_3xHA | CAATTACACCAAAAAGTAAAATTCACAAGCTTATGTACCCTTACGATGTTCCGGATTACGCATATCCCTATGACGTGCCAGACTATGCTtacccatacgatgtccctgattatgccGTAACTGGTGGCATGGCGTCCAAGTGGGACCAGAAGG |
| FCU_for | catacgatgtccctgattatgccGTAACTGGTGGCATGGCGTCCAAGTGG |
| FUR1_CDS_rev | ACTGAGCGATTCGCCTCGCATCGAATTCCTGCAGGGGCCCATAGGTGTGGCCTAGCTTGC |
| FUR1_UTR_for | CAACCTACTTACACACACCTTTCCGCTTAACCCGCTAGCCTTTCCACCCAGCGCGGGTGCATTC |
| FUR1_UTR_rev | CGTAATCCGGAACATCGTAAGGGTACATAAGCTTGTGAATTTTACTTTTTGGTGTAATTG |

Blue – FCY1 gene, green – FUR1 gene, orange – 3xHA tag
